# Isolated adrenocorticotropic hormone deficiency and thyroiditis associated with nivolumab therapy in a patient with advanced lung adenocarcinoma: a case report and review of the literature

**DOI:** 10.1186/s13256-019-2002-2

**Published:** 2019-03-26

**Authors:** Nobumasa Ohara, Michi Kobayashi, Kazumasa Ohashi, Ryo Ito, Yohei Ikeda, Gen Kawaguchi, Yuichiro Yoneoka, Go Hasegawa, Toshinori Takada

**Affiliations:** 1 Department of Endocrinology and Metabolism, Uonuma Institute of Community Medicine, Niigata University Medical and Dental Hospital, 4132 Urasa, Minamiuonuma, Niigata, 949-7302 Japan; 2 Department of Diabetes, Endocrinology and Metabolism, Center Hospital of the National Center for Global Health and Medicine, Tokyo, Japan; 3 Department of Respiratory Medicine, Uonuma Institute of Community Medicine, Niigata University Medical and Dental Hospital, Niigata, Japan; 4 Department of Radiology, Uonuma Institute of Community Medicine, Niigata University Medical and Dental Hospital, Niigata, Japan; 5 Department of Neurosurgery, Uonuma Institute of Community Medicine, Niigata University Medical and Dental Hospital, Niigata, Japan; 6 Department of Pathology, Uonuma Institute of Community Medicine, Niigata University Medical and Dental Hospital, Niigata, Japan

**Keywords:** Human leukocyte antigen, Hydrocortisone, Isolated adrenocorticotropic hormone deficiency, Lung adenocarcinoma, Nivolumab, Thyroiditis

## Abstract

**Introduction:**

Immune checkpoint inhibitors are a promising class of anticancer drugs. The clinical benefits afforded by immune checkpoint inhibitors can be accompanied by immune-related adverse events that affect multiple organs, and endocrine immune-related adverse events include thyroiditis and hypophysitis. Hypophysitis is less frequent and has a less severe clinical presentation in patients treated with other immune checkpoint inhibitors, such as nivolumab, pembrolizumab, and atezolizumab, than in those treated with ipilimumab. However, studies have described isolated adrenocorticotropic hormone deficiency cases associated with nivolumab, pembrolizumab, and atezolizumab therapy, most of which occurred during the course of immune checkpoint inhibitor therapy. We report a rare case of patient with isolated adrenocorticotropic hormone deficiency that occurred after nivolumab therapy.

**Case presentation:**

A 69-year-old Japanese woman with advanced lung adenocarcinoma developed painless thyroiditis with transient elevations of serum thyroid hormones during 3 months of cancer treatment with nivolumab and began thyroid hormone replacement therapy for subsequent primary hypothyroidism. Four months after nivolumab therapy was discontinued, she developed isolated adrenocorticotropic hormone deficiency; corticosteroid replacement therapy relieved her secondary adrenal insufficiency symptoms, such as anorexia and fatigue. Human leukocyte antigen typing revealed the presence of DRB1*04:05-DQB1*04:01-DQA1*03:03 and DRB1*09:01-DQB1*03:03-DQA1*03:02 haplotypes, which increase susceptibility to autoimmune polyendocrine syndrome associated with thyroid and pituitary disorders in the Japanese population.

**Conclusions:**

Our patient developed thyroiditis during cancer treatment with nivolumab and subsequently exhibited isolated adrenocorticotropic hormone deficiency 4 months after discontinuing the drug. Administration of nivolumab in combination with a genetic predisposition to polyglandular autoimmunity probably caused both the thyroiditis and hypophysitis, resulting in primary hypothyroidism and isolated adrenocorticotropic hormone deficiency, respectively, in our patient. The present case highlights the need for physicians to be aware that endocrine immune-related adverse events, including hypophysitis, can occur more than several months after discontinuing a drug.

## Introduction

Immune checkpoint inhibitors (ICIs) are a promising new class of anticancer drugs that reactivate cytotoxic T cells which, in turn, destroy tumor cells [1]. Because ICIs generate dysimmune toxicities (autoimmunity) by creating an imbalance within the immune system, the clinical benefits afforded by ICIs can be accompanied by immune-related adverse events (IRAEs) that affect multiple organs, mainly the skin, gut, liver, lung, and endocrine tissues [2]. Common endocrine IRAEs include thyroiditis and hypophysitis, whereas uncommon IRAEs include adrenalitis and type 1 diabetes mellitus [3].

Compared with those treated with ipilimumab, an anti-cytotoxic T lymphocyte antigen 4 monoclonal antibody, hypophysitis is less frequent and has a less severe clinical presentation in patients treated with other ICIs, such as nivolumab and pembrolizumab, which are anti-programmed cell death protein 1 (PD-1) monoclonal antibodies, and atezolizumab, an anti-programmed cell death ligand 1 (PD-L1) monoclonal antibody [4–6]. However, studies have described isolated adrenocorticotropic hormone deficiency (IAD) cases associated with nivolumab, pembrolizumab, and atezolizumab therapy [7–22], most of which have occurred during the course of ICI therapy.

IAD is a pituitary disorder characterized by secondary adrenal insufficiency (AI) with low or absent cortisol production but normal secretion of pituitary hormones other than adrenocorticotropic hormone (ACTH) [23]. Patients with IAD typically present with anorexia, fatigue, and general weakness, but corticosteroid replacement therapy is effective for managing this disorder.

We report a case of a patient with advanced lung adenocarcinoma (LAC) who developed thyroiditis during nivolumab therapy and subsequently exhibited IAD 4 months after the discontinuation of nivolumab. We also review previously reported cases of nivolumab-related thyroiditis and IAD.

## Case presentation

**Fig. 1:**
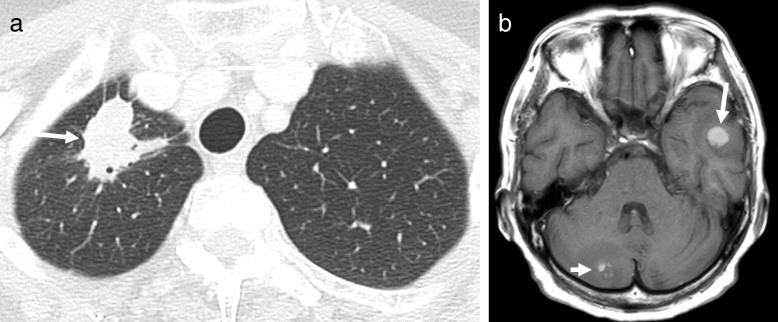
Radiological findings (July 2016). **a** Chest computed tomographic scan showing a tumor (3.1 cm) in the upper lobe of the right lung (*arrow*). **b** T1-weighted transverse magnetic resonance imaging of the brain showing a tumor in the left temporal lobe (*arrow*) and a tumor in the right cerebellar hemisphere (*short arrow*)

**Fig. 2:**
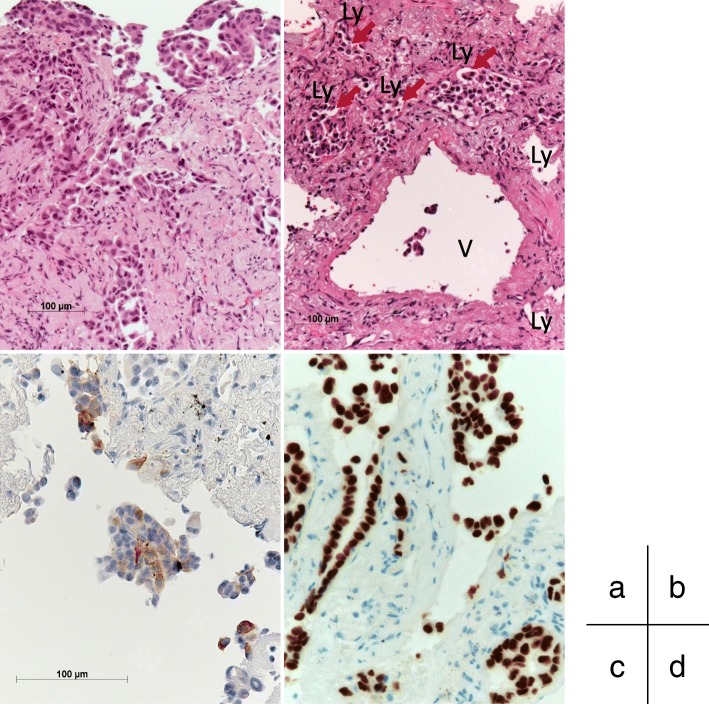
Histopathological findings of biopsy specimen from the left lung tumor (July 2016). **a**, **b** Microscopic examination showing bronchial mucosal infiltration by poorly differentiated lung adenocarcinoma (**a**), and vascular invasion is seen (*arrows*) (**b**) (H&E staining). **c**, **d** The cytoplasm of the tumor cells was immunohistochemically positive for surfactant protein A (**c**), and the tumor cell nuclei were positive for thyroid transcription factor 1 (**d**). *Ly* Lymph duct, *V* Vein

A 69-year-old Japanese woman who had been undergoing cancer treatment for advanced LAC was admitted to our hospital in January 2018 because of anorexia, fatigue, and general weakness. The patient had a maternal family history of esophageal cancer. The patient had been a housewife since her 20s, had never smoked cigarettes, and did not have a drinking habit. The patient’s medical history was unremarkable until June 2016, when an abnormal x-ray shadow was found in her right lung. A computed tomographic (CT) scan revealed a tumor (3.1 cm) in the upper lobe of her right lung (Fig. 1a), right hilar and mediastinal lymph node swellings, and liver tumors. A transbronchoscopic biopsy from the lung tumor revealed LAC with vascular invasion (Fig. 2). IHC revealed no anaplastic lymphoma kinase rearrangement, and a genetic analysis of the cancer cells detected no epidermal growth factor receptor mutation. Whole-body technetium-99m methylene diphosphonate scintigraphy revealed multiple lesions at the thoracic and lumbar vertebrae, sternum, ilium, and right ischial bones. Brain magnetic resonance imaging (MRI) revealed tumors in the left temporal lobe and right cerebellar hemisphere (Fig. 1b). As a result, the patient was diagnosed with LAC with distant metastases to the brain, liver, and bones (cT2aN2M1b, stage IV) [24].

The patient underwent stereotactic radiation surgery (total, 22 Gy) for her metastatic brain tumors in July 2016. Thereafter, she received four courses of chemotherapy with intravenous cisplatin, pemetrexed, and bevacizumab from July 2016 to October 2016; this treatment regimen effectively controlled her advanced LAC with a Response Evaluation Criteria in Solid Tumors (RECIST) classification of partial response [25]. The patient subsequently received nine courses of maintenance chemotherapy with intravenous pemetrexed and bevacizumab from November 2016 to April 2017. CT scans performed in May 2017 revealed no progression of the primary LAC or metastatic brain and bone lesions, but they showed evidence of progression of the metastatic liver tumors.

**Fig. 3:**
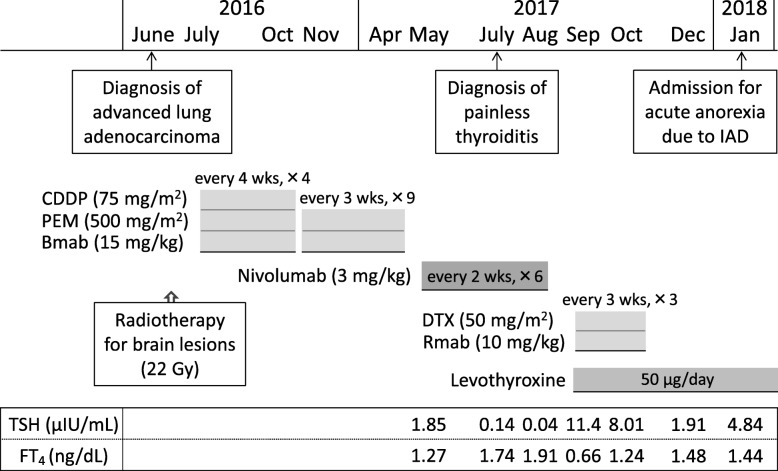
Clinical course of the patient before the onset of isolated adrenocorticotropic hormone deficiency. *Bmab* Bevacizumab, *CDDP* Cisplatin, *DTX* Docetaxel, *FT*_*4*_ Free thyroxine, *IAD* Isolated adrenocorticotropic hormone deficiency, *PEM* Pemetrexed, *Rmab* Ramucirumab, *TSH* Thyroid-stimulating hormone

**Fig. 4:**
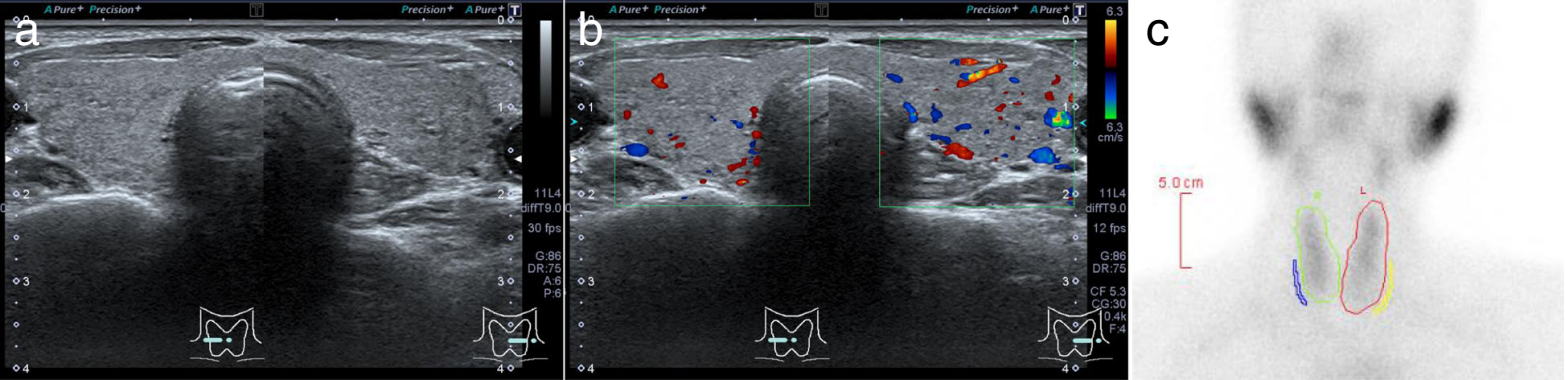
Thyroid gland imaging findings (July 2017). **a**, **b** Ultrasonography of the thyroid gland showing rough and mildly low echogenicity in a slightly enlarged thyroid gland without a tumor (**a**). Color Doppler images revealed no increased blood flow in the thyroid gland (**b**). **c** Technetium-99m pertechnetate thyroid scintigraphy showing a low thyroid uptake (0.1%, reference range 0.5–4%) in the entire thyroid gland without a hot spot

Subsequently, the patient began second-line chemotherapy with intravenous nivolumab (133 mg [3 mg/kg] every 2 weeks) in May 2017 (Fig. 3). Thyroid function was routinely monitored in July 2017 after five courses of nivolumab therapy, and she showed high levels of serum free thyroxine (FT_4_, 1.91 ng/dl) and low levels of thyroid-stimulating hormone (TSH, 0.04 μIU/ml). The patient had no symptoms of thyrotoxicosis or exophthalmos but had mild and soft struma without any pain or fever. Ultrasonography revealed rough and mildly low echogenicity in a slightly enlarged thyroid gland without a tumor (Fig. 4a, b), and technetium-99m pertechnetate thyroid scintigraphy revealed a low thyroid uptake of 0.1% (reference range, 0.5–4%) in the entire thyroid gland (Fig. 4c). The patient had negative test results for TSH-binding inhibitory immunoglobulin (TBII), thyroglobulin autoantibody (TgAb), and thyroid peroxidase autoantibody (TPOAb). On the basis of these findings, she was diagnosed with painless thyroiditis induced by nivolumab and was closely followed without medication. The patient exhibited primary hypothyroidism (FT_4_, 0.66 ng/dl; TSH, 11.41 μIU/ml) in September 2017 and initiated thyroid hormone replacement therapy with oral levothyroxine (50 μg/day).

Because the CT scans performed after the sixth cycle of nivolumab revealed enlargements of the metastatic liver tumors, nivolumab therapy was discontinued in August 2017. The patient received three courses of third-line chemotherapy with docetaxel and ramucirumab beginning in September 2017, which effectively controlled her LAC (RECIST classification of partial response), but this treatment was terminated in October 2017 because of side effects, such as joint pain and rhabdomyolysis. The patient did not consent to continue anticancer drug therapy after considering the potential benefits and side effects, and she chose to receive best supportive care.

In November 2017, the patient’s body weight, blood pressure, and pulse rate were 45 kg, 121/67 mmHg, and 76 beats per minute, respectively. She had normal levels of serum electrolytes (sodium 140 mEq/L, potassium 4.3 mEq/L, and chloride 106 mEq/L). However, the patient developed acute anorexia, fatigue, and general weakness in December 2017 and was admitted to the Department of Respiratory Medicine at our hospital in January 2018.

On admission, the patient had clear consciousness and did not complain of headache, abdominal pain, diarrhea, or joint and muscle pain. Her height, body weight, body temperature, blood pressure, and pulse rate were 153 cm, 38 kg, 36.9 °C, 90/54 mmHg, and 73 beats per minute, respectively. She had mild and soft goiter without pain. No heart murmur, chest rales, rash, vitiligo, skin pigmentation, or peripheral edema was detected. No paralysis, cerebellar ataxia, pyramidal or extrapyramidal tract symptoms, visual disturbance, hearing loss, dysarthria, or epileptic seizures were found. A blood analysis revealed hyponatremia (serum sodium 124 mEq/L); asymptomatic hypoglycemia (fasting plasma glucose 65 mg/dl); and low levels of immunoreactive insulin (< 0.2 μU/ml), ACTH (2.6 pg/ml), and cortisol (< 0.2 μg/dl) (Table 1). Because AI was suspected, oral levothyroxine was discontinued on day 2 of admission because hormone replacement therapy with thyroid hormone alone can exaggerate AI symptoms when hypothyroidism and AI coexist. The patient was referred to the Department of Endocrinology and Metabolism on day 5 of admission.

**Table 1:** Laboratory findings at admission (January 2018)

Hematology		
Red blood cells	379 × 10^4^/μl	(386–492)
Hemoglobin	11.3 g/dl	(11.6–14.8)
Hematocrit	33.7%	(35.1–44.4)
White blood cells	3600/μl	(3300–8600)
Platelets	20.9 × 10^4^/μl	(15.8–34.8)
Blood chemistry		
Fasting plasma glucose	65 mg/dl	(70–109)
Immunoreactive insulin	< 0.2 μU/ml	(2.2–12.4)
Glycated hemoglobin	5.2%	(4.6–6.2)
Total cholesterol	161 mg/dl	(142–248)
Triglycerides	62 mg/dl	(30–117)
Total protein	6.2 g/dl	(6.6–8.1)
Albumin	3.7 g/dl	(4.1–5.1)
Aspartate aminotransferase	31 IU/L	(13–30)
Alanine aminotransferase	12 IU/L	(7–23)
Creatine phosphokinase	59 IU/L	(41–153)
Urea nitrogen	11.4 mg/dl	(8.0–18.4)
Creatinine	0.66 mg/dl	(0.46–0.79)
Uric acid	2.5 mg/dl	(2.6–5.5)
Sodium	124 mEq/L	(137–147)
Potassium	4.8 mEq/L	(3.5–4.7)
Chloride	90 mEq/L	(98–108)
Calcium	8.7 mg/dl	(8.8–10.1)
C-reactive protein	0.16 mg/dl	(0–0.14)
Immunoglobulin G4	35 mg/dl	(5–117)
Carcinoembryonic antigen	208.0 ng/ml	(0–4.76)
Sialyl-Lewis X	26.6 U/ml	(0–37.9)
Cytokeratin 19 fragments	1.6 ng/ml	(0–2.1)
Squamous cell carcinoma antigen	1.1 ng/ml	(0–1.5)
Plasma osmolality	254 mOsm/L	(275–290)
Arginine vasopressin	1.0 pg/ml	
Thyroid-stimulating hormone	4.84 μIU/ml	(0.50–5.00)
Free thyroxine	1.44 ng/dl	(0.90–1.70)
Free triiodothyronine	3.22 pg/ml	(2.30–4.00)
Adrenocorticotropic hormone	2.6 pg/ml	(7.2–63.3)
Serum cortisol	< 0.2 μg/dl	(4.5–21.1)
Dehydroepiandrosterone sulfate	< 20 ng/ml	(120–1330)
Plasma renin activity	0.2 ng/ml/h	(0.2–2.3)
Aldosterone	8.8 ng/dl	(3.0–15.9)

The reference range for each parameter is shown in parentheses

Blood samples were taken in the morning (10 a.m.) with the patient in supine position. The patient was taking thyroid hormone replacement therapy with oral levothyroxine (50 μg/day) for primary hypothyroidism

**Fig. 5:**
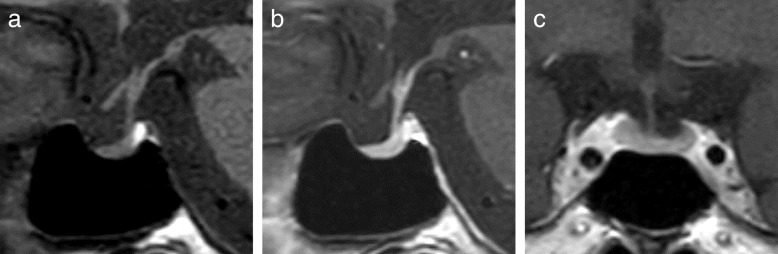
Magnetic resonance imaging of the pituitary gland (January 2018). **a** Sagittal T1-weighted plain magnetic resonance imaging (MRI) study showing normal high-intensity signals in the posterior lobe of the pituitary. **b**, **c** Gadolinium-enhanced MRI scans (**b**, sagittal plane; **c**, coronal plane) showing homogeneous enhancement of the normal hypophyseal stalk and mild atrophy of the anterior lobe of the pituitary gland. The width, length, and height of the pituitary gland are 16.2, 9.8, and 2.2 mm, respectively

A rapid cosyntropin stimulation test suggested secondary AI (Table 2). Dynamic tests assessing the secretion of pituitary hormones showed the normal release of growth hormone (GH), TSH, and prolactin; age-appropriate release of luteinizing hormone and follicle-stimulating hormone; but no ACTH release following a corticotropin-releasing hormone load (Table 3). A GH-releasing peptide 2 loading test also showed no ACTH release, whereas GH release was sufficient (Table 4). These findings were indicative of IAD. A brain MRI study revealed slight atrophy of the anterior pituitary with a pituitary height of 2.2 mm (Fig. 5).

**Table 2:** Endocrinological investigation: rapid cosyntropin stimulation test in January 2018 (day 6 after admission)

	Time (min)		
	0	30	60
Cortisol (μg/dl)	< 0.2	1.0	1.4
Aldosterone	5.7	14.1	16.5

Blood samples were taken at 0 (just before) and at 30 and 60 min after synthetic adrenocorticotropic hormone 1–24 (cosyntropin hydroxide 0.25 mg) was intravenously administered in the morning (9 a.m.). The patient had a low plasma adrenocorticotropic hormone level (2.3 pg/ml) and low plasma renin activity (< 0.2 ng/ml/h) just before cosyntropin administration

**Table 3:** Endocrinological investigation: CRH/GRF/TRH/LHRH stimulation test in January 2018 (day 7 after admission)

	Time (min)					
	0	15	30	60	90	120
Adrenocorticotropic hormone (pg/ml)	2.1	3.5	4.0	3.4	2.9	2.2
Cortisol (μg/dl)	< 0.2	< 0.2	< 0.2	< 0.2	< 0.2	< 0.2
Thyroid-stimulating hormone (μIU/ml)	5.47	22.86	33.95	28.66	23.64	17.65
Growth hormone (ng/ml)	7.67	19.96	34.68	38.76	37.53	21.17
Prolactin (ng/ml)	22.6	75.6	94.4	84.6	61.2	44.6
Luteinizing hormone (mIU/ml)	10.4	21.8	30.9	45.3	47.9	43.1
Follicle-stimulating hormone (mIU/ml)	32.8	36.2	33.6	36.6	45.6	45.5

The following synthetic hypothalamic hormones were intravenously administered in the morning (9 a.m.): human corticotropin-releasing hormone (CRH; 100 μg), growth hormone-releasing factor (GRF; 100 μg), thyrotropin-releasing hormone (TRH; 500 μg), and luteinizing hormone-releasing hormone (LHRH; 100 μg)

The hormone replacement therapy with oral levothyroxine (50 μg/day) for primary hypothyroidism was discontinued on day 2 after admission, and the patient’s serum levels of free thyroxine (1.26 ng/dl) and free triiodothyronine (2.77 pg/ml) were measured just before the drug administration

**Table 4:** Endocrinological investigation: GHRP-2 stimulation test in January 2018 (day 8 after admission)

	Time (min)				
	0	15	30	45	60
Adrenocorticotropic hormone (pg/ml)	1.9	2.3	1.9	1.9	3.2
Cortisol (μg/dl)	< 0.2	< 0.2	< 0.2	< 0.2	< 0.2
Growth hormone (ng/ml)	3.73	18.02	29.63	32.98	26.21

Growth hormone-releasing peptide (GHRP)-2 (100 μg) was intravenously administered in the morning (9 a.m.)

The patient had negative test results for anti-pituitary cell antibody, TgAb, TPOAb, and TBII as well as other organ-specific autoantibodies, including glutamic acid decarboxylase autoantibody, insulin autoantibody, gastric parietal cell autoantibody, intrinsic factor autoantibody, adrenocortical autoantibody, antinuclear antibody, Sjögren’s syndrome A and B antibodies, anti-citrullinated peptide antibody, and rheumatoid factor.

Human leukocyte antigen (HLA) typing revealed the presence of A*08:01/12:02, B*48:01/52:01, and C*08:01/12:02 class I genes and DRB1*04:05/09:01, DQB1*03:03/04:01, DQA1*03:02/03:03, and DPB1*04:02/14:01 class II genes.

The patient began corticosteroid replacement therapy with oral hydrocortisone (15 mg/day) for AI secondary to IAD on the afternoon of day 8 of admission. Subsequently, she resumed oral levothyroxine (50 μg/day) for primary hypothyroidism on day 9 of admission.

The patient experienced improvements in anorexia, fatigue, and general weakness and became ambulatory within days. Her hyponatremia was corrected within 1 week, and her hypoglycemia was resolved. Laboratory data obtained on day 21 of admission showed normal levels of serum sodium (140 mEq/L), potassium (4.3 mEq/L), chloride (106 mEq/L), FT_4_ (1.60 ng/dl), TSH (3.28 μIU/ml), and fasting plasma glucose (78 mg/dl). The patient was discharged on day 25 of admission.

The subsequent clinical course of the patient was mostly uneventful for 6 months after discharge. As her LAC progressed, her ability to perform activities of daily living decreased, and in November 2018, she was transferred to a local hospital to receive terminal care.

## Discussion

Our patient with advanced LAC developed painless thyroiditis associated with a transient elevation of serum thyroid hormones (thyrotoxicosis) during 3 months of second-line chemotherapy with nivolumab and began thyroid hormone replacement therapy for subsequent primary hypothyroidism. Four months after nivolumab therapy was discontinued, the patient developed IAD. Other than reports regarding nivolumab, there have been few reports on thyroid or pituitary dysfunction associated with the anticancer agents that our patient took, including bevacizumab and ramucirumab (Fig. 3) [26–29]. External brain radiotherapy can produce pituitary dysfunction that is usually associated with GH deficiency, but it seldom causes IAD [30]. These findings suggest that our patient exhibited both ICI-related thyroiditis and IAD induced by nivolumab.

Table 5 summarizes the characteristics of reported patients who exhibited nivolumab-related thyroiditis and IAD [15, 16, 19, 20]. The cases describe both male and female adults with melanoma, LAC, or renal cell carcinoma. They developed thyroiditis that resulted in hypothyroidism and subsequently developed IAD.

**Table 5.** Summary of reported patients who exhibited thyroiditis and isolated adrenocorticotropic hormone deficiency in association with cancer treatment with nivolumab

Ref.	Age/sex	Nivolumab			Thyroiditis				IAD				Other IRAEs
		Target cancer	Regimen	Administration times (courses)	Time from first nivolumab administration to thyroiditis onset (months)	Thyroid function abnormality	Thyroid autoantibodies	Abnormality on thyroid ultrasonography	Time from first nivolumab administration to IAD onset (months)	Major symptoms	Pituitary autoantibodies	Morphological abnormality in the pituitary on MRI	
[15]	60/M	LAC	3 mg/kg every 2 weeks	11	5	Hypothyroidism	N/D	N/D	7	Fatigue, anorexia, dizziness, gait instability	N/D	None	None
[16]	54/M	RCC	2 mg/kg every 2 weeks	12	3	Hypothyroidism	Positive (TgAb and TPOAb)	N/D	6	Fatigue, hypoglycemia	N/D	None	None
[19]	58/M	Melanoma	3 mg/kg every 2 weeks	N/D	4	Transient thyrotoxicosis and subsequent hypothyroidism	Positive (TgAb)	N/D	8	Fatigue, anorexia, weakness	N/D	None	Hypercalcemia
[20]	63/F	Melanoma	2 mg/kg every 3 weeks	8	6	Transient thyrotoxicosis and subsequent hypothyroidism	Positive (TgAb)	Low echogenicity	8	Fatigue	N/D	None	None
Present case	69/F	LAC	3 mg/kg every 2 weeks	6	2	Transient thyrotoxicosis and subsequent hypothyroidism	Negative	Low echogenicity	7	Fatigue, anorexia, weakness	Negative	Atrophy	None

*F* female, *IAD* isolated adrenocorticotropic hormone deficiency, *IRAE* immune-related adverse event, *LAC* lung adenocarcinoma, *M* male, *MRI* magnetic resonance imaging, *N/D* not described, *RCC* renal cell carcinoma, *TgAb* thyroglobulin autoantibody, *TPOAb* thyroid peroxidase autoantibody

Thyroiditis is the most frequent IRAE associated with endocrine organs, regardless of the different ICIs [3, 6]. Thyroiditis usually occurs within a few months of initiating ICI therapy with or without verifiable circulating thyroid autoantibodies, such as TgAb and TPOAb. Common clinical manifestations of ICI-related thyroiditis include painless thyroiditis characterized by a transient thyrotoxicosis and subsequent occurrence of persistent primary hypothyroidism [31, 32]. In our patient, physical findings, thyroid gland image findings (Fig. 4), and the clinical course (Fig. 3) indicated that she had a typical form of ICI-related thyroiditis.

Studies on patients with IAD associated with nivolumab have shown that the latency from initial nivolumab administration to IAD onset ranged approximately from 4 to 8 months, with a mean period of roughly 6 months [7–20], which appears to be longer than that of ipilimumab-related hypophysitis (approximately 2 months) [5]. Most cases of nivolumab-related IAD occurred during the course of or within 1 month of the discontinuation of nivolumab therapy [7–19], but one patient with melanoma who completed 6 months of nivolumab therapy 2 months prior developed IAD during subsequent ipilimumab therapy [20]. Similarly, our patient with LAC developed IAD 4 months after the discontinuation of 3 months of nivolumab therapy. Our patient’s case highlights the need for physicians to be aware that endocrine IRAEs, including hypophysitis, can occur more than several months after discontinuing a drug.

Patients with ipilimumab-related hypophysitis usually exhibit pituitary enlargement as radiological evidence of pituitary inflammation and pituitary dysfunction with multiple deficits in anterior pituitary hormones [4, 5, 33]. By contrast, many studies of nivolumab-related IAD have reported no morphological pituitary abnormalities [8–17, 19, 20], whereas two studies described patients who showed mild or slight but definitely reversible pituitary enlargement [7, 18]. However, there was a reported case of a patient with atezolizumab-related IAD who showed anterior pituitary atrophy [22]. Similarly, our patient exhibited mild anterior pituitary atrophy (Fig. 5).

Autoimmune polyendocrine syndromes (APS) are a heterogeneous group of endocrine and nonendocrine organ-specific autoimmune disorders [34]. For example, autoimmune thyroid diseases can be associated with other endocrine disorders as part of APS type 3. Genetic factors, including HLA class II genes, play a role in the development of APS. Our patient with ICI-related thyroiditis and IAD had the HLA-DRB1*04:05-DQB1*04:01-DQA1*03:03 and DRB1*09:01-DQB1*03:03-DQA1*03:02 haplotypes, which increase susceptibility to APS type 3 associated with pituitary disorders in the Japanese population [35]. These findings suggest that administration of nivolumab in combination with a genetic predisposition to polyglandular autoimmunity caused thyroiditis and hypophysitis in association with APS type 3, resulting in primary hypothyroidism and IAD, respectively, in our patient.

## Conclusions

We describe a patient with advanced LAC who developed thyroiditis during 3 months of nivolumab therapy and subsequently exhibited IAD 4 months after the discontinuation of nivolumab. The combination of nivolumab administration and genetic factors that manifested a susceptibility to polyglandular autoimmunity likely caused her ICI-related thyroiditis and hypophysitis. Thus, physicians should be aware that ICI-related endocrinopathies, including hypophysitis, may occur more than several months after discontinuation of the drug.

## Abbreviations

ACTH: Adrenocorticotropic hormone
AI: Adrenal insufficiency
APS: Autoimmune polyendocrine syndrome
Bmab: Bevacizumab
CDDP: Cisplatin
CRH: Corticotropin-releasing hormone
CT: Computed tomography
DTX: Docetaxel
F: Female
FT_4_: Free thyroxine
GH: Growth hormone
GHRP: Growth hormone-releasing peptide
GRF: Growth hormone-releasing factor
HLA: Human leukocyte antigen
IAD: Isolated adrenocorticotropic hormone deficiency
ICI: Immune checkpoint inhibitor
IRAE: Immune-related adverse event
LAC: Lung adenocarcinoma
LHRH: Luteinizing hormone-releasing hormone
Ly: Lymph duct
M: Male
MRI: Magnetic resonance imaging
N/D: Not described
PD-1: Anti-programmed cell death protein-1
PD-L1: Anti-programmed cell death ligand-1
PEM: Pemetrexed
RCC: Renal cell carcinoma
RECIST: Response Evaluation Criteria in Solid Tumors
Rmab: Ramucirumab
TBII: Thyroid-stimulating hormone-binding inhibitory immunoglobulin
TgAb: Thyroglobulin autoantibody
TPOAb: Thyroid peroxidase autoantibody
TRH: Thyrotropin-releasing hormone
TSH: Thyroid-stimulating hormone
V: Vein
